# Altered expression of signalling lymphocyte activation molecule receptors in T-cells from lupus nephritis patients—a potential biomarker of disease activity

**DOI:** 10.1093/rheumatology/kex078

**Published:** 2017-04-05

**Authors:** Victoria Stratigou, Anne F. Doyle, Francesco Carlucci, Lauren Stephens, Valentina Foschi, Marco Castelli, Nicola McKenna, H. Terence Cook, Liz Lightstone, Thomas D. Cairns, Matthew C. Pickering, Marina Botto

**Affiliations:** 1 Department of Medicine, Imperial College London, Centre for Complement and Inflammation Research; 2 Imperial Lupus Centre, Imperial College NHS Healthcare Trust, London, UK

**Keywords:** systemic lupus erythematosus, T cell, nephritis, rituximab

## Abstract

**Objectives.** The aim was to investigate whether the signalling lymphocyte activation molecule (SLAM) signalling pathways contribute to LN and whether SLAM receptors could be valuable biomarkers of disease activity.

**Methods.** Peripheral blood mononuclear cells from 30National Research Ethics Service SLE patients with biopsy-proven LN were analysed by flow cytometry. Clinical measures of disease activity were assessed. The expression of the SLAM family receptors on T-cell subpopulations [CD4, CD8 and double negative (DN) T cells] was measured and compared between lupus patients with active renal disease and those in remission.

**Results.** The frequency of CD8 T cells expressing SLAMF3, SLAMF5 and SLAMF7 was significantly lower in LN patients who were in remission. In contrast, these subsets were similar in patients with active renal disease and in healthy individuals. Patients with active nephritis had an increased percentage of circulating monocytes, consistent with a potential role played by these cells in glomerular inflammation. Changes in the frequency of DN T cells positive for SLAMF2, SLAMF4 and SLAMF7 were observed in lupus patients irrespective of the disease activity. We detected alterations in the cellular expression of the SLAM family receptors, but these changes were less obvious and did not reveal any specific pattern. The percentage of DN T cells expressing SLAMF6 could predict the clinical response to B-cell depletion in patients with LN.

**Conclusion.** Our study demonstrates altered expression of the SLAM family receptors in SLE T lymphocytes. This is consistent with the importance of the SLAM-associated pathways in lupus pathogenesis.

Rheumatology key messages

- SLE patients with inactive nephritis have fewer CD8 T cells expressing SLAMF3, SLAMF5 or SLAMF7.
- SLAMF2-, SLAMF4- and SLAMF7-positive double negative T cells are differentially expressed in lupus patients.
- There is an increased proportion of SLAMF6 double negative T cells in LN patients non-responding to B-cell depletion therapy.

## Introduction

SLE is an autoimmune multisystem inflammatory disease that is debilitating and can cause life-threatening organ damage, such as LN. Patients with SLE develop pathogenic autoantibodies that are directed towards a broad range of ubiquitous self-antigens, including dsDNA and nuclear debris from apoptotic cells. These immune complexes (ICs) deposit in organs, and the subsequent influx of inflammatory cells contributes to widespread tissue damage. Owing to its multifactorial aetiology, the immunopathogenesis of SLE is extremely complex and incompletely understood. However, it is widely accepted that loss of B-cell tolerance, in conjunction with CD4 T-cell hyperactivation, is central to lupus pathogenesis. In this context, the signalling lymphocyte activation molecule (SLAM) family receptors have recently emerged as key players in the immune dysregulation underlying lupus pathogenesis [1, 2]. Studies using knockout mice deficient in SLAM family receptors and SLAM-associated protein (SH2D1A) have shown that these molecules play an important role in T-cell-mediated help for humoral immunity [3, 4] a key process in lupus pathogenesis.

The SLAM gene family comprises functionally related cell-surface type I transmembrane receptors, which are expressed broadly on haematopoietic cells, including T, B and NK cells, myeloid and plasmacytoid dendritic cells, macrophages and monocytes. Members of this family are as follows: CD150 (SLAMF1), CD48 (SLAMF2), CD229 (SLAMF3, Ly-9), CD244 (SLAMF4, 2B4), CD84 (SLAMF5), NTB-A (SLAMF6) and CD319 (SLAMF7, CRACC, CS1). With the exception of CD48, which interacts with CD244, all of the SLAM members interact via homophilic interactions. The SLAM receptors have immunoreceptor tyrosine-based switch motifs in their intracellular domain that can be bound by SH2D1A and Ewing’s sarcoma-activated transcript 2 (SH2D1B). These interactions trigger important immunomodulatory effects [5, 6].

The SLAM gene cluster has been genetically associated with SLE in humans and in murine models of SLE. In humans, it is located within a chromosomal region, 1q23, that has been shown to have a strong linkage with SLE in genome-wide association studies [7]. Likewise, the syntenic linkage region on mouse chromosome 1 has been shown to be a genetic susceptibility region for the development of SLE in several spontaneous lupus-prone models [8]. For example, polymorphisms in the Ly108 gene (corresponding to SLAMF6 in humans) result in the generation of a Ly108 splice variant in lupus-prone mice that is involved in the pathogenesis of SLE [9]. Thus, emerging data provide strong arguments that these genes play an important role in key processes believed to lead to the development of SLE and therefore represent strong functional candidates.

LN is one the most serious clinical manifestations of SLE. It is a relapsing remitting GN that is treated with CS and immunosuppressive medications, all with a considerable side-effect burden for the patients. Thus, there is growing recognition of the need to identify marker(s) of disease flare/remission that will facilitate patient stratification according to prognosis and treatment. We explored whether expression of SLAM receptors on different T-cell subpopulations [CD4, CD8 and double negative (DN) T cells] could be used as a biomarker for LN. We analysed SLE patients with active LN and those in remission but with a history of LN. Unexpectedly, we found that patients who were clinically inactive displayed more noticeable changes in the expression of the SLAM gene family than the healthy individuals. Taken together, our data provide further support for the concept that SLAM molecules have important disease-modifying effect(s) in SLE.

## Methods

### Patients

Thirty SLE patients (all of whom met the revised ACR criteria [10] and the SLICC criteria [11]) with biopsy-proven LN were recruited from the Imperial Lupus Centre (Table 1). The LN subsets were categorized according to the International Society of Nephrology/Renal Pathology Society classification. Patients who had received CYC and/or B-cell depletion within 6 months were excluded. The BILAG index was used for clinical assessment and response. Active LN was defined as urine protein:creatinine ratio >50 mg/mmol together with biopsy-proven class III or IV or V LN within 3 months of recruitment. Inactive LN was defined as patients with a history of biopsy-proven LN and on a prednisolone dose of ⩽10 mg daily together with renal BILAG domain grade D, protein:creatinine ratio <20 mg/mmol and no change in SLE-related medication within 12 months before recruitment. The healthy volunteer cohort consisted of 4 males and 16 females, with a median age of 34 years (range 24–54 years). Samples from patients and healthy volunteers were collected as a sub-collection registered with the Imperial College Healthcare Tissue Bank (National Research Ethics Service approval 12/WA/0196). Informed consent was obtained from all contributing individuals according to the Declaration of Helsinki. The Tissue Management Committee of the Imperial College Healthcare Tissue Bank approved the application (ref R13010a) to use these samples in this study.

**Table 1:** Baseline characteristics of study cohort

Number	LN class	Current treatment	Previous CYC (total dose, g)	Previous RTX (total cycle number)	**Protein: creatinine ratio**^a^** (mg/mmol)**	SLEDAI	BILAG/ renal	SLE (LN) duration (years)
Active								
1	III (A/C)	MMF, HCQ	0	0	290	8	13/A	0 (0)
2	IV-S (A)	MMF, HCQ, Pred	0	0	223	19	45/A	8 (7)
3	III (A)	HCQ	0	0	289	13	23/A	0 (0)
4	V + III (A)	MMF, HCQ, Pred	0	1	604	18	26/B	1 (1)
5	V + III (A/C)	MMF, HCQ, Pred	0	0	77	8	17/B	1 (0)
6	V	MMF, HCQ, Pred	0	1	270	8	8/B	8 (8)
7	V + III (C)	MMF, HCQ	0	1	732	6	8/B	5 (4)
8	III (C)	MMF, HCQ, Pred	3	1	91	8	8/B	12 (10)
9	V	MMF, HCQ, Pred	6	1	134	12	13/A	30 (30)
10	V + III (A)	MMF, HCQ	0	0	2010	7	34/A	0 (0)
11	IV-S (A/C)	MMF, HCQ, Pred	0	0	161	13	31/B	6 (0)
12	III (A/C)	HCQ, Pred, i.v.MP×3	0	0	83	17	15/C	8 (0)
13	V + IV-S (A/C)	AZA, Pred	6	1	1236	10	13/A	6 (5)
14	IV-G (A)	MTX, HCQ, Pred, i.m.MP×1	0	1	211	13	30/B	3 (3)
15	V + IV-G (A/C)	MMF, Pred	3	0	272	8	12/A	17 (17)
16	V + IV-G (C)	MMF, HCQ	0	1	398	4	12/A	7 (7)
17	IV−G (C)	HCQ, Pred	0	0	161	4	14/A	25 (24)
18	V	MMF	3	0	294	8	12/A	17 (13)
19	III (A)	MMF, HCQ	3	1	229	8	13/A	18 (12)
Inactive								
20	IV	AZA, Pred	0	0	<20	0	0/D	35 (35)
21	IV	Pred	3	0	<20	0	0/D	28 (26)
22	V + III (C)	MMF, HCQ	0	1	<20	0	0/D	29 (28)
23	V	MMF	0	1	<20	0	0/D	11 (10)
24	III (A)	AZA, HCQ	0	0	<20	4	0/D	15 (15)
25	III	HCQ	0	0	<20	2	1/D	10 (10)
26	IV	HCQ	3	1	<20	0	0/D	4 (4)
27	V	AZA, HCQ	0	0	<20	2	1/D	30 (25)
28	III	AZA, HCQ	0	1	<20	3	1/D	7 (7)
29	III	None	0	1	<20	2	2/D	8 (8)
30	IV-G (A)	AZA, HCQ	0	3	<20	6	2/D	11 (7)

CYC and RTX refer to treatments given at least 6 months before this study. LN duration was calculated from the time of the first renal biopsy.

a The protein:creatinine ratio normal range is <20 mg/nmol. MP: methylprednisolone; Pred: prednisolone; RTX: rituximab.

Ten out of the 19 active LN patients were treated with rituximab (anti-CD20 antibody). The rituximab regimen was two doses of rituximab (1 g) and methylprednisolone (500 mg) on days 1 and 15 [12]. In two patients, methylprednisolone was omitted because of concomitant oral prednisolone. Owing to rituximab hypersensitivity, one patient received ofatumumab (a fully humanized anti-CD20 antibody). Background immunosuppressive medications were continued if taken at baseline. Renal response to B-cell depletion at 12 months was defined as follows: protein:creatinine ratio decrease by >50% from baseline, estimated glomerular filtration rate ⩾60 ml/min or if <60 ml/min at baseline, not fallen by >20% and improvement in overall disease activity scores (SLEDAI/BILAG). Patients not meeting these criteria at 1 year post B-cell depletion were defined as non-responders. Non-responders included patients who required treatment escalation.

### Lymphocyte analysis by flow cytometry

Peripheral blood mononuclear cells, isolated from whole blood by density gradient centrifugation, were stained using the following panels: blood cell populations (efluor450 anti-CD3, FITC anti-CD4, APC-efluor 780 anti-CD8, PE-CY7 anti-CD19, PerCP-efluor 710 anti-CD14, PE anti-CD16 and APC anti-CD56); B-cell phenotype (efluor450 anti-CD3, PE-CY7 anti-CD19, PE anti-CD24, APC anti-CD27, FITC anti-CD38 and PerCP-efluor 710 anti-IgD); NK and NK T cells (APC or PE anti-CD56, PerCP-efluor 710 anti-CD16, efluor-450 anti-CD3, PE anti-Nkp30, PerCP-efluor 710 anti-Nkp44, APC anti-Nkp46, PE-CY7 anti-αβTCR and FITC anti-γδTCR); T-cell phenotype [PE-CY7 anti-CD4, APC-efluor 780 anti-CD8, V500 anti-CD3 (BD Biosciences, San Diego, CA, USA), PerCP-efluor 710 anti-CCR7, FITC anti-CD45RA, efluor-450 anti-CD45RO and PE anti-CXCR5]. SLAM receptor expression on T cells was determined as follows: V500 anti-CD3, PE-CY7 anti-CD4, APC-efluor 780 anti-CD8, PE anti-CD150, BV421 anti-CD48 (Biolegend, San Diego, CA, USA), APC anti-CD229, FITC anti-CD244, APC anti-CD84, PE anti-CD352 and PE anti-CD319. Gate strategies are illustrated in supplementary Fig. S1, available at *Rheumatology* Online. All antibodies were obtained from e-Bioscience (San Diego, CA, USA) unless noted differently. Non-specific Fc-mediated interactions were blocked with human Fc receptor binding inhibitor. Flow cytometry was performed with a BD FACSVerse (BD Biosciences). Data were analysed using FlowJo software, version 10 (TreeStar, Ashland, OR, USA).

### Statistical analysis

Results were expressed as the mean (s.d.) or median with interquartile range. Comparisons between two groups were performed using the Mann–Whitney *U*-test, between more than two groups using the analysis of variance with Tukey’s multiple comparison test. A value of P < 0.05 was considered statistically significant. Statistical analysis was performed using GraphPad Prism version 7.0 (GraphPad Software, San Diego, CA, USA).

## Results

### Baseline clinical characteristics and cellular phenotyping

A cohort of 30 biopsy-proven LN patients was evaluated. Demographic features and baseline clinical scores are shown in Table 1. The median age of the patients was 34.5 years, and the disease duration ranged from 0 to 35 years (8 years). Only three patients were males. Among the 30 LN patients, 19 had active LN and 11 had inactive LN. Median age and disease duration as well as the distribution of sex and ethnic background were similar in the active and inactive LN patients. The LN histological classes were also equally distributed between the two groups.

We initially assessed the distribution of the peripheral blood subpopulations (Table 2). As expected, lupus patients displayed several cellular changes compared with the healthy controls. The most notable differences included a significant decrease in the frequency of NK cells; in particular, of the CD56^Dim^ NK fraction among the NK cells; and a strikingly increased percentage of naïve B cells. These changes were independent of the disease activity at the time of the analysis.

**Table 2:** Flow cytometry analysis of peripheral blood mononuclear cells

Cell population (%)	Flow cytometry staining	Group			P-value		
		A	I	HD	A *vs* I	A *vs* HD	I *vs* HD
		(n = 19)	(n = 11)	(n = 21)			
Total CD3	CD3^+^	47.84 (16.87)	53.70 (16.17)	56.19 (10.22)	0.5303	0.1669	0.8872
CD4	CD3^+^ CD4^+^	38.23 (20.50)	33.82 (18.40)	45.83 (14.55)	0.7909	0.3750	0.1757
Central memory CD4	CD3^+^ CD4^+^ CD45RA^−^ CCDR7^+^	18.74 (9.36)	28.38 (5.99)	32.75 (13.90)	0.0915	0.0008*	0.5918
Effector CD4	CD3^+^ CD4^+^ CD45RO^+^	8.24 (5.85)	1.72 (0.91)	7.22 (6.61)	0.0185*	0.8435	0.0514
Naïve CD4	CD3^+^ CD4^+^ CD45RA^+^ CCDR7^+^	29.73 (14.86)	39.34 (15.76)	26.51 (9.66)	0.1990	0.7235	0.0592
Follicular CD4	CD3^+^ CD4^+^ CXCR5^+^	8.5 (4.60)	9.14 (4.47)	13.43 (4.97)	0.9321	0.0058*	0.0500*
CD8	CD8^+^ CD8^+^	29.98 (17.01)	20.98 (14.66)	23.46 (11.17)	0.2350	0.3415	0.8910
Central memory CD8	CD3^+^ CD8^+^ CD45RA^−^ CCDR7^+^	3.16 (5.21)	3.87 (1.45)	4.56 (4.94)	0.9237	0.6166	0.9277
Effector CD8	CD3^+^ CD8^+^ CD45RO^+^	27.59 (13.42)	19.59 (17.63)	27.98 (14.11)	0.3705	0.9961	0.3307
Naïve CD8	CD3^+^ CD8^+^ CD45RA^+^ CCDR7^+^	37.92 (21.12)	56.92 (31.18)	31.49 (13.32)	0.0698	0.5977	0.009
DN	CD3^+^ CD4^−^ CD8^−^	5.75 (3.43)	3.68 (1.77)	5.25 (3.34)	0.1945	0.8688	0.3786
Ratio CD4/CD8		1.49 (0.74)	2.23 (1.77)	2.27 (1.04)	0.2173	0.0959	0.9945
NK	CD3^−^ CD56^+^	6.57 (3.26)	6.23 (4.84)	14.12 (7.94)	0.9873	0.0006*	0.0023*
CD56^Dim^ NK	CD3^−^ CD56^Dim^ CD16^Bright^	59.05 (15.22)	58.05 (27.18)	88.7 (5.77)	0.9853	<0.0001*	<0.0001*
CD56^Bright^ NK	CD3^−^ CD56^Bright^ CD16^Low^	7.86 (4.95)	9.45 (11.04)	2.62 (2.63)	0.7810	0.0333*	0.0157*
NKT	CD3^+^ CD56^+^	1.51 (1.66)	1.17 (1.08)	2.85 (3.81)	0.9396	0.2754	0.2284
Total B	CD3^−^ CD19+	6.97 (7.23)	4.42 (1.73)	7.09 (11.42)	0.7123	0.9989	0.6850
Early memory B	CD3^−^ CD19^+^ IgD^+^ CD27^+^	3.37 (3.88)	8.57 (10.91)	17.19 (12.90)	0.3578	0.0002*	0.0634
Late memory B	CD3^−^ CD19^+^ IgD^−^ CD27^+^	17.67 (14.07)	20.57 (22.78)	35.36 (27.16)	0.9359	0.0407*	0.1838
Naïve B	CD3^−^ CD19^+^ IgD^+^ CD27^−^	66.39 (21.78)	66.61 (34.92)	1.83 (1.73)	0.9996	<0.0001*	<0.0001*
Plasma cells	CD3^−^ CD19^+^ IgD^−^ CD27^+^ CD38^+^	20.17 (19.26)	8.99 (9.10)	5.27 (4.78)	0.0700	0.0023*	0.7279
Transitional B	CD3^−^ CD19^+^ IgD^High^ CD27^−^ CD38^High^ CD24^High^	8.48 (7.10)	3.7 (5.14)	5.29 (3.99)	0.0725	0.1880	0.7301
Monocytes	CD14^+^	17.18 (8.75)	11.52 (3.59)	9.80 (3.13)	0.0422*	0.0011*	0.7285

Results are expressed as the mean (±s.e.m.). The P-values were considered statistically significant when P < 0.05 according to one-way analysis of variance with Tukey’s multiple comparison test.

*Significant P-values. A: SLE patients with active LN; DN: double negative T cells; HD: healthy donors; I: SLE patients with inactive LN; NKT: natural killer T cells; n: total number of patients in the group.

Patients with active LN had an increased frequency of plasma cells, whereas the percentages of early memory B cells, follicular and central memory CD4 T cells were reduced compared with those in healthy donors. Of note, an increased monocyte proportion was the only feature that distinguished active LN from both inactive LN and controls (Table 1 and supplementary Fig. S2, available at *Rheumatology* Online). This relative increase is likely to be the result of the more severe lymphopenia in patients with active disease.

### SLAM receptors on DN and CD8 T cells—potential biomarkers of renal disease activity

**Fig. 1:**
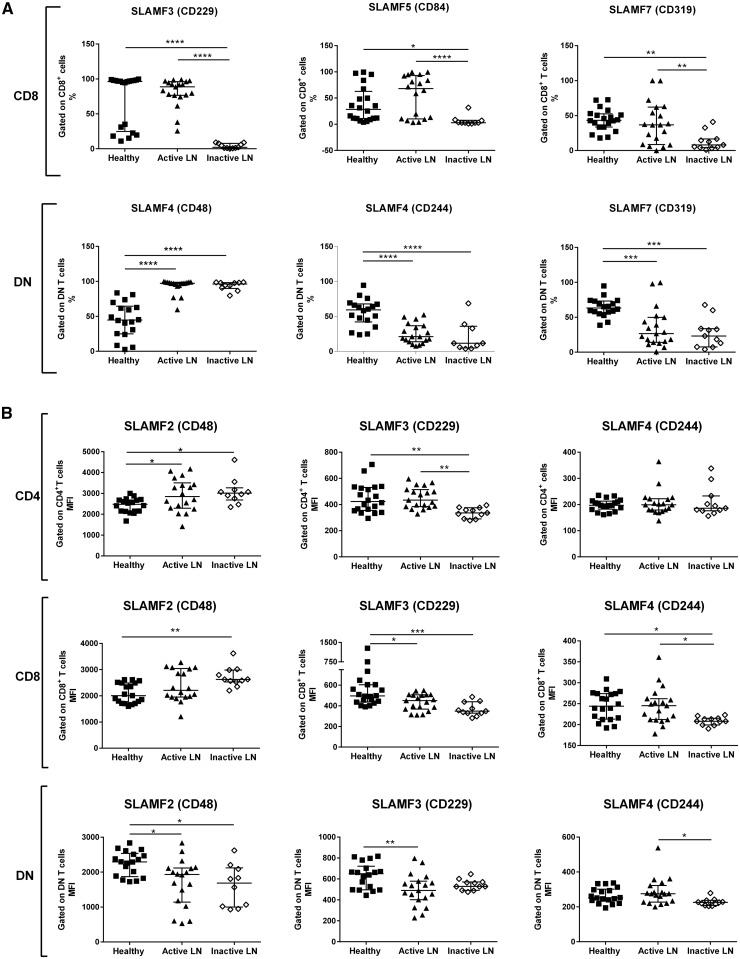
Cellular analysis of signalling lymphocyte activation molecule receptors on T cells Total T cells from 20 healthy donors and 19 active and 11 inactive LN patients were analysed for surface SLAM receptor expression by flow cytometry. (**A**) Frequency of SLAM receptor-positive T cells. (**B**) Mean fluorescence intensity of SLAM receptors. Bars represent median with interquartile range. P-values were determined by one-way analysis of variance with Tukey’s multiple comparison test: *P < 0.05, **P < 0.005, ***P < 0.0005 and ****P < 0.0001. DN: double negative; MFI: mean fluorescence intensity; SLAM: signalling lymphocyte activation molecule.

Previous reports have shown that the SLAM gene family may act as an important alternative pathway for T-cell co-stimulation and that certain members are expressed abnormally in peripheral blood mononuclear cells from SLE patients [13–16]. To assess this in our patient cohort, we analysed all SLAM receptors on the three main T-cell subpopulations: CD4, CD8 and DN cells. Owing to technical limitations, we aborted the assessment of SLAMF1 expression after the analysis of the first 12 patients. At this stage, there were no differences between the three experimental groups (data not shown). The study of the remaining SLAM members, SLAMF2–SLAMF7 inclusive, is presented in Table 3, and the most informative findings are shown in Fig. 1. The most prominent differences were noted in the percentages of DN and CD8 T cells expressing SLAM receptors. The frequency of DN T cells positive for SLAMF2, SLAMF4 or SLAMF7 was markedly altered in SLE patients, but these differences were unrelated to the disease activity. In contrast, the proportion of CD8 T cells expressing SLAMF3, SLAMF5 or SLAMF7 was significantly lower in the lupus patients in clinical remission compared with the other two groups (Fig. 1A). A repeated analysis using samples taken at a different time from a small number of individuals showed consistent results, demonstrating that the changes were stable (data not shown). Differences in the expression of SLAMF2, SLAMF3 or SLAMF4 were also noticed, but these changes were less obvious and did not show a clear pattern (Fig. 1B). Overall, in comparison with healthy controls, the differences in expression were more marked in the inactive rather than the active LN patients.

**Table 3:** Analysis of signalling lymphocyte activation molecule receptors on CD4^+^, CD8^+^ and double negative T cells

		Frequency of positive cells						Intensity of expression					
SLAM	Cell population	%			P-values			MFI			P-values		
		A	I	HD	A *vs* I	A *vs* HD	I *vs* HD	A	I	HD	A *vs* I	A *vs* HD	I *vs* HD
		(n = 19)	(n = 11)	(n = 20)				(n = 19)	(n = 11)	(n = 20)			
SLAMF2 (CD48)	CD4^+^	73.38 (32.44)	65.31 (23.55)	82.89 (20.15)	0.7185	0.5290	0.2191	2919 (783.7)	3091 (635.0)	2421 (348.1)	0.7579	0.0490*	0.0220*
	CD8^+^	52.02 (28.78)	43.58 (28.60)	49.64 (24.57)	0.7124	0.9626	0.8390	2381 (594.2)	2718 (383.3)	2094 (376.8)	0.1609	0.1660	0.0031*
	DN	93.03 (10.82)	93.66 (6.30)	44.95 (25.17)	0.9954	<0.0001*	<0.0001*	1709 (679.3)	1609 (600.1)	2249 (351.6)	0.8925	0.0148*	0.0148*
SLAMF3 (CD229)	CD4^+^	91.71 (15.20)	78.68 (14.18)	88.32 (18.27)	0.0991	0.7940	0.2668	451.8 (80.46)	337.1 (39.86)	448.3 (111.5)	0.0037*	0.9915	0.0046*
	CD8^+^	81.14 (20.27)	3.93 (3.45)	70.85 (37.22)	<0.0001*	0.4607	<0.0001*	556.8 (198.7)	435.6 (79.88)	366.1 (64.60)	0.3911	0.0241*	0.0018*
	DN	73.66 (23.15)	59.27 (12.86)	76.62 (24.77)	0.2059	0.9123	0.1094	495.8 (153.4)	544.1 (51.22)	630.1 (122.1)	0.5684	0.0058*	0.1822
SLAMF4 (CD244)	CD4^+^	1.97 (2.66)	1.39 (1.90)	0.96 (1.57)	0.7486	0.3073	0.8555	206.6 (48.66)	209.9 (57.56)	198.2 (21.63)	0.9766	0.8099	0.7422
	CD8^+^	37.08 (23.10)	22.14 (20.49)	41.38 (18.32)	0.1489	0.7951	0.0442*	244.8 (43.43)	208.6 (9.97)	245.2 (33.75)	0.0223*	0.9994	0.0195*
	DN	25.04 (14.18)	20.53 (22.0)	55.67 (19.66)	0.5410	<0.0001*	<0.0001*	285.9 (77.06)	226.5 (21.38)	265.2 (44.47)	0.0249*	0.2729	0.1578
SLAMF5 (CD84)	CD4^+^	56.41 (9.49)	48.39 (16.88)	51.83 (13.56)	0.2708	0.5271	0.7746	366 (99.88)	336.3 (88.91)	399.5 (91.58)	0.7058	0.5240	0.2056
	CD8^+^	54.71 (38.80)	6.49 (9.17)	37.70 (33.53)	0.0015*	0.1148	0.0341*	472.2 (202.6)	494.9 (382.1)	466.8 (135.1)	0.9657	0.9971	0.9462
	DN T	76.06 (14.17)	72.27 (14.19)	65.89 (12.33)	0.7577	0.0722	0.4595	432.3 (130.7)	345.9 (62.67)	432.5 (143.1)	0.1986	1	0.1974
SLAMF6 (CD352)	CD4^+^	52.34 (23.27)	29.26 (22.39)	36.59 (26.70)	0.0459*	0.1356	0.7117	1617 (429.4)	1324 (205.4)	1545 (559.4)	0.2193	0.8819	0.4059
	CD8^+^	55.55 (22.05)	31.62 (31.97)	45.05 (28.63)	0.0660	0.4744	0.4003	1976 (516.6)	1600 (211.7)	1950 (695.7)	0.1862	0.9885	0.2251
	DN	65.96 (19.14)	47.37 (31.08)	56.69 (39.71)	0.2740	0.6476	0.7163	1943 (476.1)	1634 (217.6)	1970 (714.8)	0.3074	0.9876	0.2494
SLAMF7 (CD319)	CD4^+^	13.35 (30.69)	2.55 (4.54)	12.72 (23.10)	0.5661	0.9353	0.5661	628.5 (426.9)	379.5 (78.29)	410.9 (144.4)	0.0588	0.0588	0.7677
	CD8^+^	39.22 (30.28)	13.13 (12.81)	43.72 (15.67)	0.0084*	0.7999	0.0016*	559.4 (469.6)	327.5 (59.54)	362 (47.23)	0.1038	0.1007	0.9477
	DN	34.61 (28.25)	27.17 (21.05)	63.94 (13.44)	0.6488	0.0006*	0.0002*	584.8 (397.9)	371.6 (75.72)	399.3 (67.50)	0.0847	0.0837	0.9576

Frequency of positive cells and intensity of expression. Data are from healthy donors and active and inactive LN patients. Results are expressed as the mean (s.e.m.). The P-values were considered statistically significant when P < 0.05 according to one-way analysis of variance with Tukey’s multiple comparison test.

*Significant P-values. A: SLE patients with active LN; HD: healthy donors; I: SLE patients with inactive LN; SLAM: signalling lymphocyte activation molecule.

### SLAMF6 expression as a potential predictor of the clinical response of LN to B-cell depletion

**Fig. 2:**
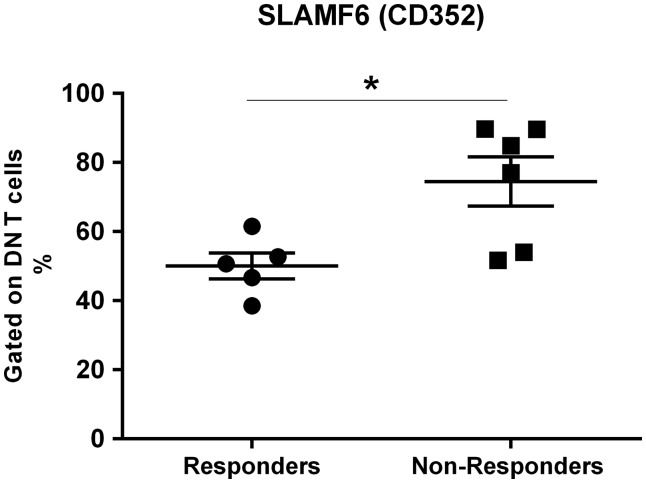
Frequency of double negative T cells expressing SLAMF6 at baseline Comparison of the percentage of SLAMF6-positive DN T cells between responders and non-responders. Response to B-cell depletion was determined at 12 months post-infusion. P-values were determined by the Mann–Whitney *U*-test: *P = 0.0303. DN: double negative; SLAM: signalling lymphocyte activation molecule.

During the course of the study, a subset of patients (n = 11) with active LN received a B-cell depletion therapy. As we had detected marked differences in the expression of the SLAM receptors on T cells between SLE patients and controls, we explored whether the SLAM cellular phenotype was correlated with the clinical response to B-cell depletion. The clinical features of this subset cohort before B-cell depletion and at 12 months are shown in Table 4. Five out of the 11 patients were classified as responders using the criteria described in the Methods section. The remaining six patients required CYC because of clinical deterioration. Having established the pattern of response among the patients, we then assessed whether the frequency of the SLAMF-positive DN T cells could have predicted the clinical outcome at baseline. Our data showed that the LN patients who failed to achieve clinical remission had a higher frequency of SLAMF6 DN T cells at baseline (P = 0.0303; Fig. 2). Consistent with the notion that these non-responders might have a more refractory renal disease, co-engagement of SLAMF6 on DN T cells has previously been shown to result in the production of greater amounts of pro-inflammatory cytokines, such as IFN-γ and TNF-α, compared with co-stimulation with anti-CD28 [13].

**Table 4:** Characteristics of the study cohort treated with B-cell depletion

Number	LN class	Baseline treatment	Rituximab		**Protein:creatinine ratio**^a^**(mg/mmol)**		SLEDAI		BILAG/renal		End point (months)
**Responders**			**Regimen**	**Cycle**^b^	**Baseline**	**+12 months**	**Baseline**	**+12 months**	**Baseline**	**+12 months**	
10	V + III (A)	MMF, HCQ	RTX + i.v. MP×2	1	2010	387	7	4	34/A	12/B	12
11	IV-S (A/C)	MMF, HCQ, Pred	RTX	1	161	<20	13	3	31/B	2/D	12
12	III (A/C)	MMF, HCQ, Pred, i.v. MP×3	RTX + i.v. MP×2	1	83	<20	17	4	15/C	3/D	12
14	IV-G (A)	MTX, HCQ, Pred, imMPx1	RTX + i.v. MP×2	2	211	90	13	6	30/B	3/C	12
13	V + IV-S (A/C)	AZA, Pred	RTX + i.v. MP×2	2	1236	189	10	3	13/A	2/C	12
**Non-responders**			**Regimen**	**Cycle**^b^	**Baseline**	**At flare**	**Baseline**	**At flare**	**Baseline**	**At flare**	**Time of flare (months)**
1	III (A/C)	MMF, HCQ	RTX + i.v. MP×2	1	290	275	8	8	13/A	22/A	3
2	IV-S (A)	MMF, HCQ, Pred	RTX	1	223	198	19	14	45/A	22/A	1
3	III (A)	HCQ	RTX + i.v. MP×2	1	289	243	12	6	23/A	14/A	3
4	V + III (A)	MMF, HCQ, Pred	Ofatumumab	2	604	382	18	22	26/A	19/A	1
19	III (A)	MMF, HCQ	RTX + i.v. MP×2	2	229	485	8	9	13/A	13/A	3
7	V + III (C)	MMF, HCQ	RTX + i.v. MP×2	3	732	1469	6	7	8/B	9/B	3

All patients received two infusions of 1000 mg of RTX or the fully humanized CD20 monoclonal ofatumumab with hydrocortisone to prevent infusion reactions. Patients defined as non-responders were treated with CYC at the time of flare.

a The protein:creatinine ratio normal range is <20 mg/nmol.

b Cycle number indicates total number of cycles received, including the one during this study. MP: methylprednisolone; Pred: prednisolone; RTX: rituximab.

## Discussion

The importance of the SLAM receptors in SLE derives from their broad expression and their immunomodulatory roles in the cross-talk between a number of immune cells [6]. We focused our analysis on T-cell expression of SLAM receptors in a cohort of SLE patients with biopsy-proven nephritis categorized into those with active and those with inactive LN. Our data showed that patients in clinical remission have markedly reduced percentages of CD8 T cells expressing SLAMF3, SLAMF5 or SLAMF7. This may indicate decreased CD8 T-cell activation. In contrast, changes in the frequency of the SLAMF2-, SLAMF4- and SLAMF7-positive DN T cells, which share a gene expression profile with the CD8 T cells, distinguished the lupus patients from healthy controls. However, these changes were independent of nephritis activity. We also found a higher proportion of CD352^+^ (SLAMF6) DN T cells in patients with active LN who subsequently failed to improve with B-cell depletion therapy. The engagement of SLAMF6 can prime T cells to produce Th1 cytokines, especially from CD8 and DN T cells [13]. We speculate that the higher frequency of SLAMF6^+^ DN T cells, through enhanced Th1 cytokine production, reflects a more aggressive LN phenotype. If confirmed, this may have clinical utility as a biomarker when deciding between B-cell depletion therapy and CYC regimens. There is a need for additional biomarkers beyond histological phenotyping, because it is clear from our data (Table 4) and others that the sub-type of LN is not correlated with treatment response [17, 18].

Unsurprisingly, in our lupus cohort we observed several cellular changes that mirror published findings. In agreement with previous reports [19, 20], we found a higher frequency of naïve B cells, defined as CD19^+^ IgD^+^ CD27^−^, in the SLE patients compared with healthy controls. This increase was irrespective of the disease activity, in keeping with the notion that even inactive SLE patients fail to remove self-reactive naïve B cells [21]. In contrast, studies have reported that the CD27^−^ naïve B-cell subpopulation is markedly reduced in the peripheral blood of lupus patients [22, 23]. The heterogeneity of CD27^−^ B cells, previously thought to represent exclusively naïve B cells, has recently become apparent, and thus the CD27^−^ population may also contain memory B cells [24]. The use of these different markers makes it difficult to compare studies. However, in the active SLE patients we observed a reduction in the percentage of antigen-experienced CD27^+^ memory B cells, a finding that is in conflict with other studies [25]. Memory B cells are less susceptible to immunosuppressive therapy because they are not highly proliferative and conventional immunosuppressive drugs depend on cell cycling. We speculate that our findings may reflect the fact that many of our patients had a long history of LN and had been treated with B-cell-depleting therapy. It might be that repetitive anti-CD20 treatment cycles results in more substantial depletion of the memory B-cell compartment compared with non-B-cell-depleting immunosuppressive regimens. Independently, memory B cells can easily differentiate into plasma cells because they have lower activation thresholds. Consistent with this, we found a significantly increased fraction of plasmablasts/plasma cells in the SLE patients with active LN. This is in agreement with reports showing strong correlations between the frequencies of plasma cells and disease activity [26] or the time of clinical relapse after B-cell-depleting therapies [27, 28]. Another striking difference between the patients and the healthy controls was the marked reduction of the total NK cell frequency. Specifically, the CD56^Dim^ fraction, which is the most abundant one, was reduced, whereas the CD56^Bright^ was slightly increased. Although numerical and functional defects in NK cells have been described previously [29–33], the NK cell lymphopenia seems to be a key feature of SLE patients with renal involvement [34]. Whether this is the result of the migration of the CD56^Dim^ NK cells, a cell type with high cytotoxic capacity, into the inflamed kidney can only be speculated without further studies at the site of tissue damage.

Several studies have explored the expression of the SLAM receptors on immune cells from SLE patients, but the results are variable. This is likely to reflect the heterogeneity of SLE. To address this, we chose to study SLE patients with biopsy-proven LN. Our cohort was well characterized and our definitions of active and inactive LN were rigorous to enable us to explore reliably whether LN disease activity and the response of LN to B-cell depletion were associated with differential expression of T-cell SLAM genes. The SLAM receptors are important alternative pathways of T-cell co-stimulation. Consequently, it is not surprising that we found several abnormalities in SLE T cells. However, the most striking abnormalities were detected in the CD8 and DN T cells rather than CD4 T cells. CD8 and DN T cells are closely linked populations, because at least some of the DN cells may represent CD8 subsets that have lost CD8 from the surface membrane [35, 36]. Although the DN T-cell population was not expanded in our SLE cohort, the proportions of SLAMF2-, SLAMF4- and SLAMF7-expressing cells were significantly different in the lupus patients, regardless of the disease activity, compared with the healthy controls. Consistent with our observation a study by Kis-Toth *et al.* [14] showed that SLE patients had significantly fewer SLAMF4-expressing CD8 T cells compared with healthy controls and that these cells were functionally impaired. Interestingly, these cells had an increased propensity to lose CD8 and to become DN T cells, spontaneously as well as upon activation. Furthermore, a reduced proportion of NK cells and monocytes positive for SLAMF4 was reported by Kim *et al.* [16], and a single nucleotide polymorphism of SLAMF4 has been associated with the presence of renal and neuropsychiatric manifestations in SLE patients [37]. SLAMF4 is known to interact with high affinity with SLAMF2 (CD48), and this interaction can mediate both activating and inhibitory pathways, depending on the cell type and the experimental conditions. It is thus intriguing that we found an increased proportion of SLAMF2-expressing DN T cells in the SLE patients, a finding that may indicate a compensatory mechanism.

Our study also revealed a striking loss of CD8 T cells expressing SLAMF3, SLAMF5 or SLAMF7 in the lupus patients in clinical remission. In keeping with the recent notion that an exhausted CD8 T-cell signature may predict good clinical outcome [38, 39], these changes may represent an exhausted cellular distribution. Previous studies on the expression and role of SLAMF3, SLAMF5 and SLAMF7 in SLE have focused on CD4 T cells, plasmacytoid dendritic cells or NK cells and highlighted how the ligation of these receptors may promote prolonged inflammation and tissue damage [15, 40]. Interestingly, it has been shown that plasmacytoid dendritic cells from SLE patients have lower expression of SLAMF5 and SLAMF7, whereas SLAMF3 is decreased on CD56^dim^ NK cells. More importantly, their expression was found to be upregulated by RNA-containing ICs, and these may trigger increased IFN-α secretion, promoting disease development [15]. Therefore, it is tempting to speculate that the lower frequency of SLAMF3-, SLAMF5- and SLAMF7-positive CD8 T cells in clinically inactive patients may reflect the reduced activation by pathogenic RNA-containing ICs. At the same time, these changes might decrease the activation threshold of these cells, acting as a negative feedback mechanism that helps to maintain the clinical remission. Further studies on the precise function of these SLAM receptors on CD8 T cells will be required to prove this formally.

The efficacy of B-depleting therapies in SLE remains controversial, with contradictory results from open-label and randomized controlled trials [41–44]. Even in studies that have demonstrated a benefit from treatment with rituximab, there has been considerable variability in initial response and time to relapse. We therefore explored whether the cellular expression of the SLAM family receptors could be a valuable predictive marker of renal response to B-cell depletion and help to apply B-cell-depleting agents more effectively. By monitoring the response at 1 year in a small cohort of LN patients, we found that patients with more circulating SLAMF6-expressing DN T cells before treatment failed to respond to B-cell depletion and required additional immunosuppressive therapy. In our study, as in a previous report [13], the expression of SLAMF6 on the surface of the T-cell subpopulations analysed was comparable between the SLE T cells and the normal T cells. However, the proportion of SLAMF6-expressing CD4 T cells was higher in the active lupus patients compared with the patients in remission. Interestingly, the SLAMF6-driven co-stimulation has been shown to be defective in SLE T cells. The defect was more pronounced in CD8 and DN T cells that also appear to produce more cytokines following engagement of SLAMF6 [13, 40]. Although it is tempting to propose that the frequency of SLAMF6^+^ DN T cells could help to identify LN patients requiring aggressive immunosuppressive regimens, such as CYC, it is also important to recognize that our finding could be relevant for only a small group of patients and thus have a limited value in clinical practice. Future studies in other LN cohorts are needed to validate the use of SLAMF6 on DN T cells as a predictive biomarker of response to B-cell depletion.

In summary, by analysing a selective group cohort of SLE patients with biopsy-proven LN we discovered several abnormalities in the SLAM family receptors on T lymphocytes. We identified a selective loss of CD8 T cells expressing SLAMF3, SLAMF5 or SLAMF7 in the lupus patients in clinical remission. In addition, we have shown that SLAMF6 on DN T cells could be a useful marker for assessing the response to B-cell depletion therapy. Overall, our study provides further support to the notion that the SLAM gene family plays a crucial role in lupus pathogenesis.

## Supplementary Material

Supplementary Data
