# Supplementary material for: Altered expression of signalling lymphocyte activation molecule receptors in T-cells from lupus nephritis patients—a potential biomarker of disease activity
Source: Rheumatology (Oxford). 2017 Apr 5;56(7):1206–16. doi: 10.1093/rheumatology/kex078 (PMC5850773; doi:10.1093/rheumatology/kex078)
Supplement: Supplementary Data [file kex078_supp.docx]

**SUPPLEMENTARY DATA**

**Supplementary Figure S1. Flow cytometric analysis of SLAMF expression**


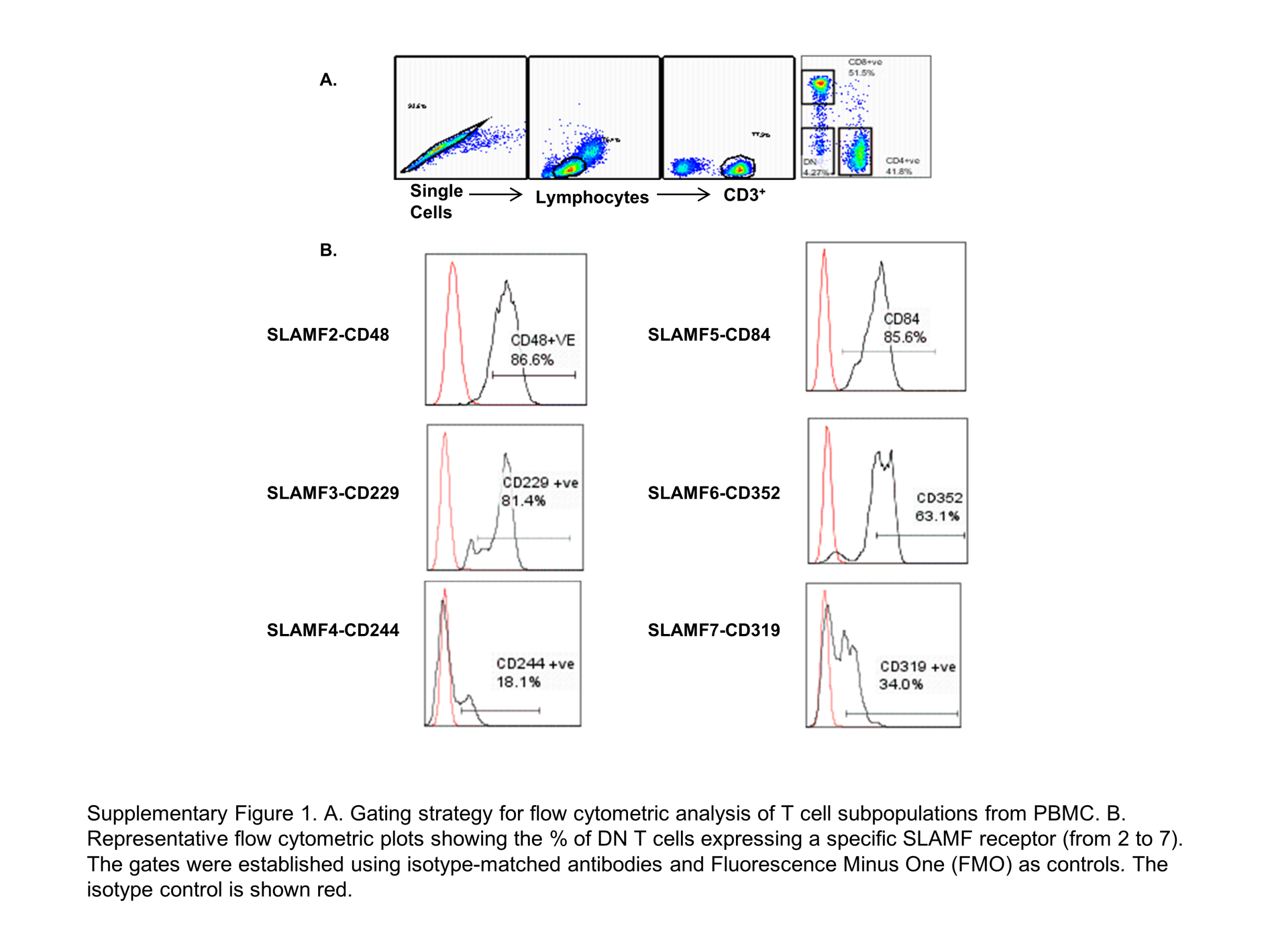
(A) Gating strategy for flow cytometric analysis of T cell subpopulations from PBMC. (B) Representative flow cytometric plots showing the % of DN T cells expressing a specific SLAMF receptor (from 2 to 7). The gates were established using isotype-matched antibodies and Fluorescence Minus One (FMO) as controls. The isotype control is shown red.

**
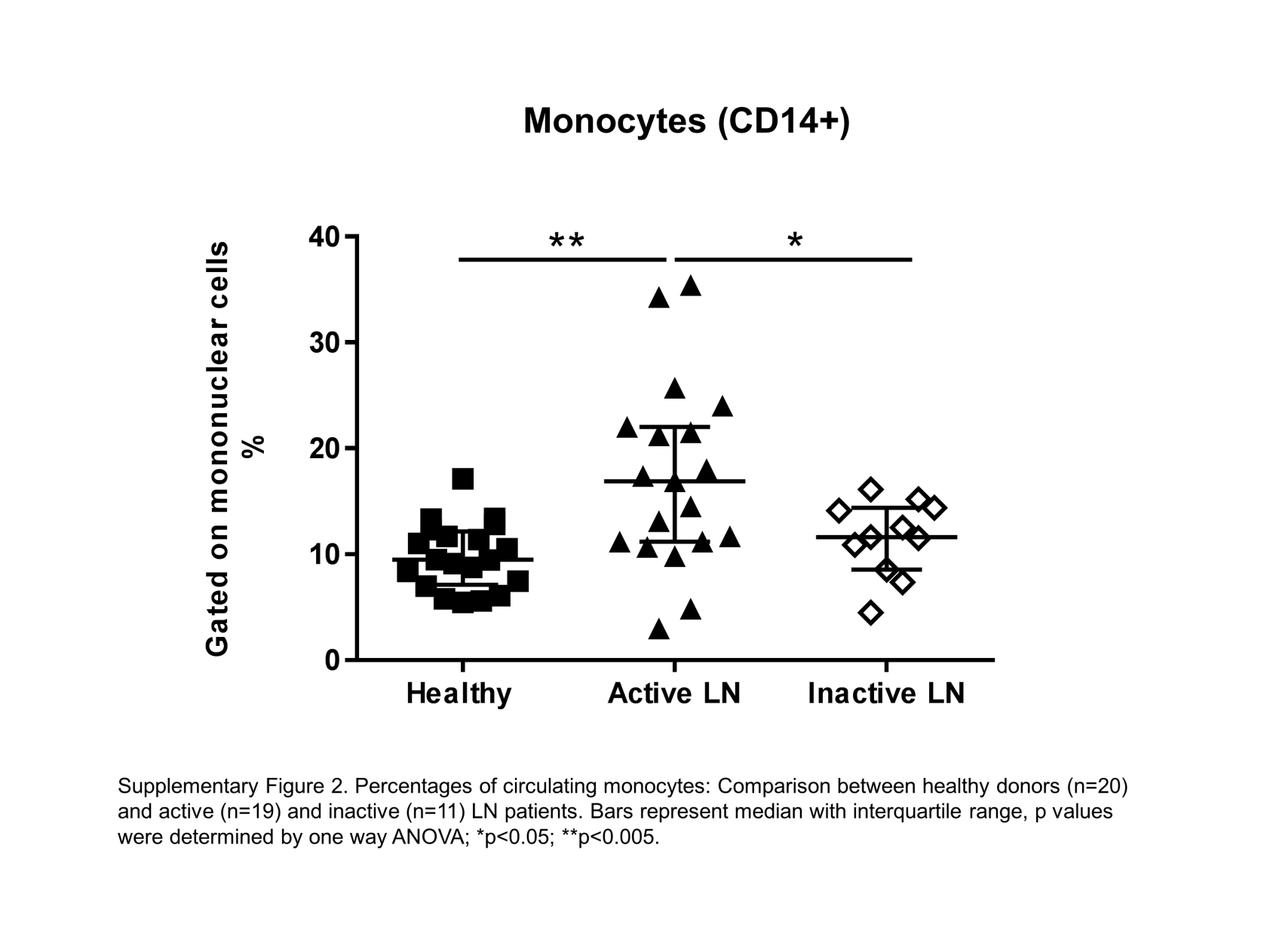
Supplementary Figure S2. Percentages of circulating monocytes**

Comparison between healthy donors (n+20) and active (n=19) and inactive (n=11) LN patients. Bars represent median with interquartile range, p values were determined by one way ANOVA; *p<0.05; **p<0.005.
